# Rodent-Borne Orthohantaviruses in Vietnam, Madagascar and Japan

**DOI:** 10.3390/v13071343

**Published:** 2021-07-12

**Authors:** Fuka Kikuchi, Kae Senoo, Satoru Arai, Kimiyuki Tsuchiya, Nguyễn Trường Sơn, Masaharu Motokawa, Marie Claudine Ranorosoa, Saw Bawm, Kyaw San Lin, Hitoshi Suzuki, Akira Unno, Keisuke Nakata, Masashi Harada, Keiko Tanaka-Taya, Shigeru Morikawa, Motoi Suzuki, Tetsuya Mizutani, Richard Yanagihara

**Affiliations:** 1 Center for Infectious Disease Epidemiology and Prevention Research, Tokyo University of Agriculture and Technology, Fuchu, Tokyo 183-8538, Japan; S203828z@st.go.tuat.ac.jp (F.K.); tmizutan@cc.tuat.ac.jp (T.M.); 2 Center for Surveillance, Immunization, and Epidemiologic Research, National Institute of Infectious Diseases, Tokyo 162-8640, Japan; ksenoo@niid.go.jp (K.S.); ktaya@nih.go.jp (K.T.-T.); mosuzuki@niid.go.jp (M.S.); 3 Faculty of Science, Tokyo University of Science, Tokyo 162-8601, Japan; 4 Laboratory of Bioresources, Applied Biology Co., Ltd., Tokyo 107-0062, Japan; 5 Institute of Ecology and Biological Resources, Vietnam Academy of Science and Technology, Hanoi 100000, Vietnam; ntson@iebr.vast.vn; 6 Graduate University of Science and Technology, Vietnam Academy of Science and Technology, Hanoi 100000, Vietnam; 7 The Kyoto University Museum, Kyoto University, Kyoto 606-8501, Japan; motokawa.masaharu.6m@kyoto-u.ac.jp; 8 Mention Foresterie et Environnement, Ecole Supérieur des Sciences Agronomiques, Université d’Antananarivo, Antananarivo 101, Madagascar; maricollaris@gmail.com; 9 Department of Pharmacology and Parasitology, University of Veterinary Science, Nay Pyi Taw 15013, Myanmar; bestshadow@gmail.com; 10 Department of Aquaculture and Aquatic Disease, University of Veterinary Science, Nay Pyi Taw 15013, Myanmar; kyawsanlinuvs@gmail.com; 11 Laboratory of Ecology and Genetics, Graduate School of Environmental Science, Hokkaido University, Kita-ku, Sapporo 060-0810, Japan; htsuzuki@ees.hokudai.ac.jp; 12 Local Independent Administrative Agency Hokkaido Research Organization, Bibai 079-0198, Japan; unno-akira@hro.or.jp (A.U.); nakata-keisuke@hro.or.jp (K.N.); 13 Laboratory Animal Center, Osaka City University, Sumiyoshi, Osaka 545-8585, Japan; haradam2651@gmail.com; 14 Department of Microbiology, Faculty of Veterinary Medicine, Okayama University of Science, Imabari 794-8555, Japan; s-morikawa@vet.ous.ac.jp; 15 Department of Pediatrics, John A. Burns School of Medicine, University of Hawaii at Manoa, Honolulu, HI 96813, USA; ryanagih@hawaii.edu

**Keywords:** Hantaan orthohantavirus, Thailand orthohantavirus, Puumala orthohantavirus, spill-over infection, rodents

## Abstract

Hantaviruses are harbored by multiple small mammal species in Asia, Europe, Africa, and the Americas. To ascertain the geographic distribution and virus-host relationships of rodent-borne hantaviruses in Japan, Vietnam, Myanmar, and Madagascar, RNA*later*™-preserved lung tissues of 981 rodents representing 40 species, collected in 2011–2017, were analyzed for hantavirus RNA by RT-PCR. Our data showed Hantaan orthohantavirus Da Bie Shan strain in the Chinese white-bellied rat (*Niviventer confucianus*) in Vietnam, Thailand; orthohantavirus Anjo strain in the black rat (*Rattus rattus*) in Madagascar; and Puumala orthohantavirus Hokkaido strain in the grey-sided vole (*Myodes rufocanus*) in Japan. The Hokkaido strain of Puumala virus was also detected in the large Japanese field mouse (*Apodemus speciosus*) and small Japanese field mouse (*Apodemus argenteus*), with evidence of host-switching as determined by co-phylogeny mapping.

## 1. Introduction

Formerly classified in the genus *Hantavirus* of the family *Bunyaviridae*, hantaviruses have been reclassified recently into four genera (*Orthohantavirus*, *Loanvirus*, *Mobatvirus,* and *Thottimvirus*) of the family *Hantaviridae* [1]. Hantaviruses possess a tripartite, negative-sense, single-stranded RNA genome comprising S, M, and L segments that encode a nucleocapsid protein, Gn and Gc envelope glycoproteins, and an RNA-dependent RNA polymerase, respectively [2,3]. Rodents (order Rodentia) have long been known to serve as reservoir hosts of hantaviruses [4]. Recently, the geographic landscape of hantaviruses has been disrupted by the identification of genetically divergent hantaviruses in multiple species of shrews and moles (order Eulipothyla) and bats (order Chiroptera) in Europe, Asia, Africa, and the Americas [5].

Several rodent-borne orthohantaviruses, notably Hantaan virus (HTNV), Seoul virus (SEOV), Puumala virus (PUUV), and Dobrava virus (DOBV), cause hemorrhagic fever with renal syndrome (HFRS) in Europe and Asia [4]. The seminal discovery of HTNV in the striped field mouse (*Apodemus agrarius*) in Korea [6] made possible subsequent discoveries of other rodent-borne hantaviruses. For example, PUUV was originally identified in the bank vole (*Myodes glareolus*), collected in the Puumala region around Lake Finland in October 1977 [7]. Genetic variants of PUUV, namely Hokkaido orthohantavirus (PUUV Hokkaido strain) and Muju orthohantavirus (PUUV Muju strain), have been identified in the grey-sided vole (*Myodes rufocanus*) in Japan [8,9,10] and the Korean red-backed vole or royal vole (*Myodes regulus*) in South Korea [11,12], respectively. 

Another rodent-borne hantavirus, Thailand orthohantavirus (THAIV), initially identified in the greater bandicoot rat (*Bandicota indica*) in Thailand [13,14], has not been definitively shown to cause HFRS, but seroepidemiological data suggest that THAIV may be pathogenic in humans [15]. Genetic variants of THAIV include Serang virus in the Asian house rat (*Rattus tanezumi*) in Indonesia [16] and Singapore [17] and Anjozorobe virus in the black rat (*Rattus rattus*) and Major’s tufted-tailed rat (*Eliurus majori*) in Madagascar [18]. Instead of the previous belief that each hantavirus species is hosted by a single mammalian host species, a single hantavirus species may be carried by multiple reservoir host species [5]. Mounting evidence suggests that preferential host switching and local adaptation account for the complex evolutionary history of hantaviruses [19,20,21].

The objective of the present study was to determine the geographic distribution and virus-host relationships of rodent-borne hantaviruses in Japan, Vietnam, Myanmar, and Madagascar. Our data showed three distinct rodent-borne orthohantaviruses: Hantaan orthohantavirus Da Bie Shan strain (HTNV DBS) in the Chinese white-bellied rat (*Niviventer confucianus*) in Vietnam; Thailand orthohantavirus Anjozorobe strain (THAIV ANJO) in the black rat in Madagascar; and Puumala orthohantavirus Hokkaido strain (PUUV HOK) in the grey-sided vole in Japan. As determined by co-phylogeny mapping, PUUV HOK infection in the large Japanese field mouse (*Apodemus speciosus*) and small Japanese field mouse (*Apodemus argenteus*) was consistent with host-switching.

## 2. Materials and Methods

### 2.1. Trapping and Sample Collection

From 2011 to 2017, small mammals were trapped at various locations in Vietnam, Myanmar, Madagascar, and Japan using Sherman live traps (H. B. Sherman Traps, Inc., Tallahassee, FL, USA). Trapped animals were euthanized according to guidelines of the American Society of Mammalogists [22,23]. Lung tissues were preserved in RNA*later*™ RNA Stabilization Reagent (Qiagen Inc., Valencia, CA, USA) and stored at −30 °C for RNA and DNA extraction. To aid in morphological identification, weight and body measurements were recorded. Field investigation procedures and protocols were approved by the Institutional Animal Care and Use Committee of the National Institute of Infectious Diseases (permission numbers: 108074, 111126, 112152, 115162, 118180).

### 2.2. RNA Extraction and RT-PCR Analysis

Total RNA was extracted from RNA*later*™-preserved lung tissues using the MagDEA RNA 100 Kit and Magtration systems 12GC PLUS (Precision System Science, Matsudo, Japan) and then reverse transcribed using PrimeScript II 1st strand cDNA Synthesis Kit (Takara Bio, Otsu, Japan) with oligonucleotide primer (OSM55F, 5′–TAGTAGTAGACTCC−3′), designed from the conserved 5′-ends of the S, M, and L segments of hantaviruses [24,25,26,27,28]. Oligonucleotide primers used to amplify the S-, M-, and L-genomic segments are provided in Supplementary Table S1. PCR cycling condition and reagent concentrations were described previously [29]. PCR products were confirmed by MultiNA with DNA-12000 kit (Shimazu, Kyoto, Japan). PCR products were treated with ExoSAP enzyme (New England Biolab, Ipswich, MA, USA) and sequenced directly using an Applied Biosystems 3730 × l DNA Analyzer (Applied Biosystems, Foster City, CA, USA).

### 2.3. Host Identification

Genomic DNAs were prepared from RNA*later*™-preserved lung tissues using the MagDEA DNA 200 and Magtration systems 12GC PLUS (PSS). DNA samples were amplified by PCR with the primer sets and sequence protocol described previously [24,30,31]. Analysis of cytochrome *b* (*CYTB*) and cytochrome *c* oxidase subunit I (*COI*) and phylogenetic relationship were conducted by ClustalW [32] in BioEdit [33] and Genetyx Ver 15 (Genetyx Corporation, Shibuya, Tokyo, Japan). 

### 2.4. Genetic and Phylogenetic Analysis

The entire and partial length of coding regions (CDS) of the S-, M-, and L-segment nucleotide and amino acid sequences of rodent-borne hantaviruses were aligned with hantavirus sequences available on GenBank, using ClustalW in BioEdit. Pair-wise comparisons were performed to ascertain the degree of sequence homology.

Phylogenetic trees were generated using the Markov chain Monte Carlo (MCMC) methods MrBayes 3.1.2 [34] under the best-fit general time-reversible model of nucleotide evolution with gamma-distributed rate heterogeneity and invariable sites (GTR + I + Γ) [35]. The best-fit model was selected with jModelTest version 2.1.7 [36] for phylogenetic trees. Two replicate Bayesian Metropolis–Hastings MCMC runs, each consisting of six chains of 10 million generations sampled every 100 generations with a burn-in of 25,000 (25%), resulted in 150,000 trees overall. In addition, to ascertain the co-evolutionary history of hantaviruses and their hosts, phylogenetic trees were reconstructed for co-phylogeny mapping [37] from a virus tree into a host tree [38].

## 3. Results

### 3.1. Trapping Surveys

**Figure 1:**
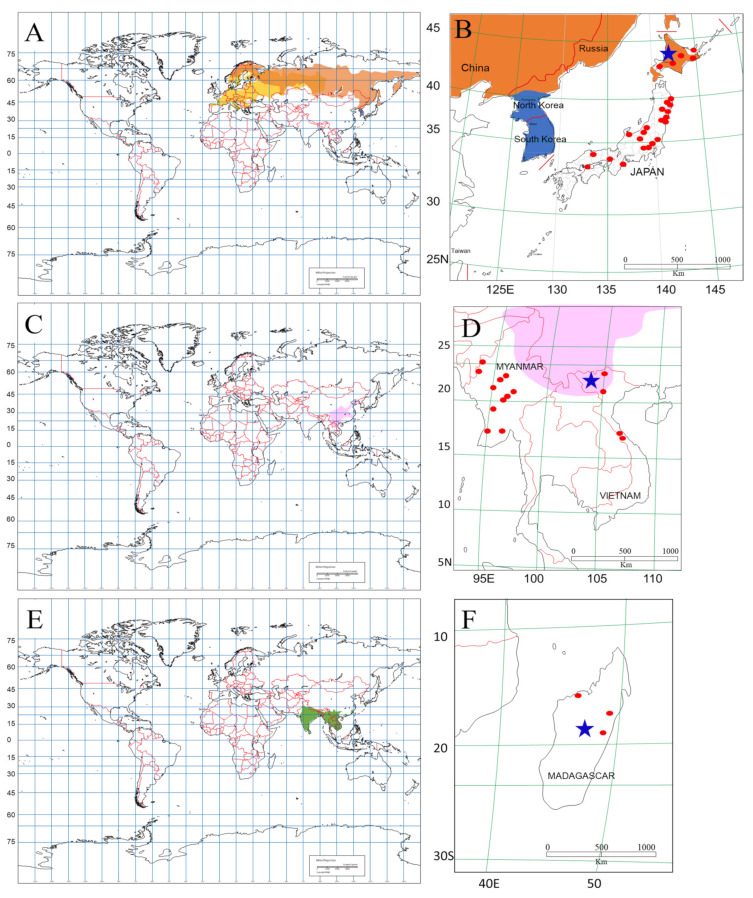
Maps showing locations of the collection sites (red circles) in Japan, Myanmar, Vietnam, and Madagascar. Hantavirus-infected rodents were captured in Japan, Vietnam, and Madagascar (blue stars, **B**,**D**,**F**). Geographic distributions of rodent host species of Pummala orthohantavirus—*Myodes glareolus* (yellow orange), *Myodes rufocanus* (orange), and *Myodes regulus* (blue)—are shown in (**A**); a host species of Hantaan orthohantavirus, *Niviventer confucianus* (pink), is distributed in northern Vietnam, eastern Myanmar, and southern China (**C**); a host species of Thailand orthohantavirus (THAIV), *Bandicota indica* (green), is distributed in South Asia (**E**).

Trapping of small mammals was conducted in Japan, Madagascar, Myanmar, and Vietnam (Figure 1). The results of field surveys are summarized in Table 1. A total of 981 mammals representing 40 species, including two species of *Apodemus*, three species of *Bandicota*, two species of *Berylmys*, one species of *Dacnomys*, three species of *Eothenomys*, one species of *Leopoldamys*, two species of *Maxomys*, one species of *Micromys*, one species of *Microtus*, seven species of *Mus*, three species of *Myodes*, five species of *Niviventer*, and seven species or species complex of *Rattus*. 

### 3.2. Hantavirus Screening and Sequence Analyses

Lung tissues were analyzed for hantavirus RNA using S-, M-, and L-segment primer sets. Sequencing of the PCR products and pair-wise alignment and comparison of the S-, M-, and L-segment nucleotide and amino acid sequences indicated that the newly detected hantaviruses were genetically similar to prototypic hantavirus species. That is, HTNV DBS strain was detected in two *Niviventer* cf. *confucianus* from Vietnam, THAIV ANJO strain in one *Rattus rattus* from Madagascar, and PUUV HOK strain in nine *Myodes rufocanus*, one *Apodemus argenteus,* and two *Apodemus speciosus* from Japan. Hantaviruses, host species, and countries are listed in Supplementary Table S2. 

### 3.3. Phylogenetic Analysis and Host Species Identification

**Figure 2:**
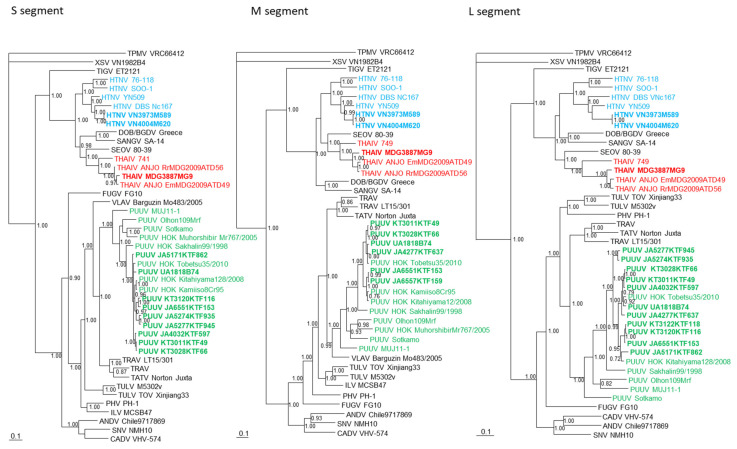
Phylogenetic trees generated by the Bayesian method, under the best-fit GTR + I+Γ model of evolution, were based on the S, M-, and L-genomic segments of Hantaan orthohantavirus (HTNV VN3973M589, VN4004M620, light blue bold) from *Niviventer* cf. *confucianus* in Vietnam, Thailand orthohantavirus (THAIV MDG3887MG9, red bold) from *Rattus ratus* species complex in Madagascar, Puumala orthohantavirus (PUUV KT3011KTF49, KT3028KTF66, KT3120KTF116, KT3122KTF118, JA4032KTF597, JA4277KTF637, JA5171KTF862, JA5277KTF945, JA6551KTF153, JA6557KTF159, green bold) from *Myodes rufocanus bedfordiae*, PUUV UA1818B74 and JA6557KTF159 (green bold) from *Apodemus speciosus*, and PUUV JA5274KTF935 (green bold) from *Apodemus argenteus* in Japan. The phylogenetic trees show the positions of representative rodent-borne hantaviruses, including Sin Nombre orthohantavirus (SNV NMH10, S: NC_005216; M: NC_005215; L: NC_005217), Cano Delgadito orthohantavirus (CADV VHV-574, S: NC_034528; M: DQ284451; L: GQ200821), Andes orthohantavirus (ANDV Chile9717869, S: AF291702; M: AF291703; L: AF291704), Prospect Hill orthohantavirus (PHV PH-1, S: Z49098; M: X55129; L: EF646763), Isla Vista orthohantavirus (ILV MC-SB-47, S: U19302; M: U19304), Tula orthohantavirus (TULV M5302v, S: NC_005227; M: NC_005228; L: NC_005226; TULV TOV.Xinjiang33, S: MN052672; M: MN183139; L: MN183135), Tatenale orthohantavirus (TATV Norton_Juxta, S: MK883757; M: MK883759; L: MK883761), Traemmersee orthohantavirus (TRAV, S: MK542662; M: MK542663; L: MK542664, LT15/301, S: MT441733; M: MT441737; L: MT441740), PUUV Sotkamo (S: NC_005224; M: NC_005223; L: NC_005225), PUUV MUJ 11–1 (S: JX028273; M: JX028272; L: JX028271), PUUV HOK Olhon 109Mrf (S: KP325674; M: KM245956; L: KM245961), PUUV HOK Sakhalian99/1998 (S: AB675453; M: AB675454; L: AB675455), HOK/Muhorshibir/Mr767/2005 (S: AM930972; M: AM930975), PUUV HOK Kitahiyama128/2008 (S: AB675463; M: AB676848; L: AB712372), PUUV HOK Tobetsu35/2010 (S: AB675450; M: AB675451; L: AB675452), HOK Kamiiso-8Cr-95 (S: AB010730; M: AB011631), Dobrava/Belgrade orthohantavirus (DOB/BGDV Greece, S: NC_005233; M: NC_005234; L: NC_005235), HTNV 76–118 (S: NC_005218; M: NC_005219; L: NC_005222), HTNV SOO-1 (S: AY675349; M: AY675353; L: DQ056292), HTNV DBS Nc167 (S: AB027523; M: AB027115; L: DQ989237), HTNV YN509 (S: GU329991; M: GU329992; L: GU329993), Sangassou orthohantavirus (SANGV SA-14, S: JQ082300; M: JQ082301; L: JQ082302), THAIV 741 (S: AB186420, 749, M: L08756; L: LC553715), THAIV ANJO EmMDG2009ATD49 (S: KC490918; M: KC490919; L: KC490922), THAIV ANJO RrMDG2009ATD56 (S: KC490916; M: KC490921; L: KC490923), Seoul orthohantavirus (SEOV 80–39, S: NC_005236; M: NC_005237; L: NC_005238), and Tigray orthohantavirus (TIGV ET2121, S: KU934010; M: KU934009; L: KU934008) as well as a bat-borne hantavirus, Xuân Sơn mobatvirus (XSV VN1982B4, S: KC688335; M: KU976427; L: JX912953), from *Hipposideros pomona* and a shrew-borne hantavirus, Thottapalayam thottimvirus (TPMV VRC66412, S: AY526097, M: EU001329, L: EU001330), from *Suncus murinus*, respectively. The numbers at each node are posterior node probabilities (>0.7) based on 150,000 trees: two replicate MCMC runs consisting of six chains of two million generations each sampled every 100 generations with a burn-in of 25,000 (25%). The scale bar indicates nucleotide substitutions per site.

**Figure 3:**
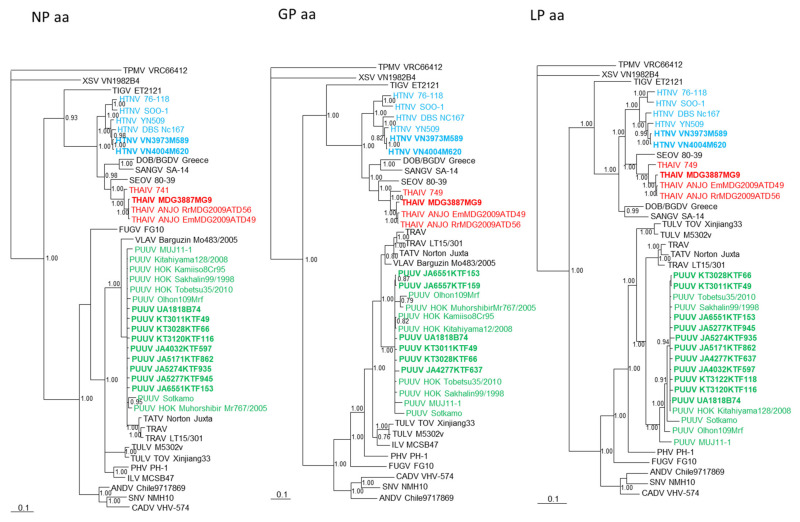
Phylogenetic trees generated by the Bayesian method, were based on the nucleocapsid protein (NP aa), glycoprotein (GP aa), and RNA-dependent RNA polymerase (LP aa) of HTNV VN3973M589 and VN4004M620 (light blue bold) from *Niviventer* cf. *confucianus* in Vietnam, THIAV MDG3887MG9 (red bold) from *Rattus rattus* species complex in Madagascar, and PUUV KT3011KTF49, KT3028KTF66, KT3120KTF116, KT3122KTF118, JA4032KTF597, JA4277KTF637, JA5171KTF862, JA5277KTF945, and JA6551KTF153 (green bold) from *Myodes rufocanus Bedfordview,* PPUV UA1818B74 and JA6557KTF159 (green bold) from *Apodemus speciosus*, and PUUV JA5274KTF935 (green bold) from *Apodemus argenteus* in Japan. Other HTNV strains are shown in light blue lettering, THAIV in red, PUUVs in green, and other representative hantaviruses in black. The numbers at each node are Bayesian posterior probabilities (>0.7) based on 15,000 trees: two replicate Markov chain Monte Carlo runs, consisting of six chains of 1 million generations each sampled every 100 generations with a burn-in of 2500 (25%). Scale bars indicate nucleotide substitutions per site.

Phylogenetic trees, constructed using the Bayesian method and based on each genomic segment of HTNV strains VN3973M589 and VN4004M620, THAIV strain MDG3887MG9, and PUUV strains UA1818B74, KT3011KTF49, KT3028KTF66, KT3120KTF116, KT3122KTF118, JA4032KTF597, JA4277KTF637, JA5171KTF862, JA5274KTF935, JA5277KTF945, JA6551KTF153, and JA6557KTF159 showed that each of the hantaviruses clustered with the prototypic hantavirus species (Figure 2 and Figure 3). 

Molecular confirmation of the rodent host species was based on the sequence analyses of the *CYTB* and *COI* genes of mitochondrial DNA. One *Niviventer* sp. was confirmed as *Niviventer* species using morphological and genetic analysis, but it was not clearly *N. confucianus* based on morphology. Therefore, we have decided to tentatively classify it as *N.* cf. *confucianus*. PUUV Hokkaido strains were confirmed as the grey-sided vole (*My. r. bedfordiae*). The subspecies, *My. r. bedfordiae,* is a geographically different species from *My. r. rufocanus*. *My. r. bedfordiae* is distributed only on Hokkaido Island and small areas in Sakhalinskaya and Far East Russia. THAIV ANJO strain was detected in *R. rattus* species complex in Madagascar. THAIV ANJO-positive rodent was captured in the Tsimbazaza Zoo in Antananarivo, Madagascar.

**Figure 4:**
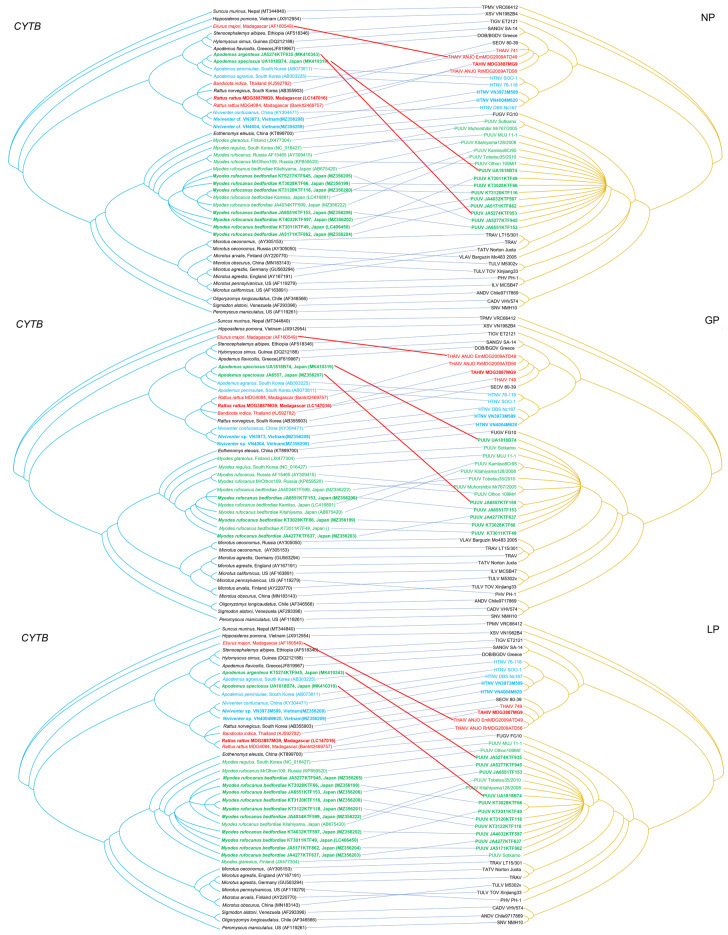
Comparisons of host mitochondria genes and hantavirus amino acid sequences generated by TreeMap 3, using Bayesian method, based on the cytochrome *b* (*CYTB*) gene of rodents (left side) and S, M, and L gene products of hantaviruses (right side). The host tree on the left was based on *CYTB* gene sequences, while the hantavirus tree on the right was based on the amino acid sequences of nucleocapsid protein (NP), glycoprotein (GP), and RNA-dependent RNA polymerase (LP), respectively. Blue lines represent expected associations between hosts and hantaviruses. Red lines indicate disparate host-hantavirus associations. Letterings for taxa are shown in green for the host species of PUUV, light blue for host species of HTNV, red for host species of THAIV, and black for the others. Newly detected hantaviruses are shown in bold lettering (light blue for VN3973M589 and VN4004M620, red for MDG3887MG9, and green for KT3011KTF49, KT3028KTF66, KT3120KTF116, KT3122KTF118, JA4032KTF597, JA4277KTF637, JA5171KTF862, JA5277KTF945, JA6551KTF153, UA1818B74, JA6557KTF159, and JA5274KTF935) in each right panel. The host, species, and virus relationships (*CYTB* and each segment sequence accession number) are listed in Supplemental Table S3.

The analysis of host-virus relationship using co-phylogeny mapping showed congruent topologies for nearly all hantaviruses and their reservoir host species (Figure 4). For example, the phylogenetic positions of PUUV strains UA1818B74, JA6557KTD158, and JA5274KTF953, based on the NP, GP, and LP, segregated with other PUUV strains and *Myodes* host species. On the other hand, the reservoir host species phylogenies of the small Japanese field mouse (*A. argenteus*) and large Japanese field mouse (*A. speciosus*) showed evidence of host switching. That is, PUUV UA1818B74, PUUV JA6557KTF159, and PUUV JA5274KTF935 had switched from its natural rodent reservoir host and become well established in a rodent host of a different genus. THAIV ANJO strain in Madagascar was also detected in the Major’s tufted-tailed rat (*E. majori*), a different host species from *R. rattus*. 

## 4. Discussion

The present study demonstrated three orthohantavirus species: one each in Japan, Vietnam, and Madagascar. PUUV was detected in *My. rufocanus*, *A. specious,* and *A. argenteus* in Hokkaido, Japan. On the other hand, three *Myodes* species (*My. rufocanus*, *M. rex,* and *M. rutilus*) are distributed in Hokkaido, Japan; we were able to detect hantavirus RNA only in *My. rufocanus*. Several research groups have conducted seroepidemiological and/or molecular epidemiological surveys nationwide in Japan thus far. PUUV is distributed in only Hokkaido, Japan [39,40]. On the other hand, *A. speciosus* and *A. argenteus* are distributed nationwide in Japan. However, PUUV has not been detected in either *Apodemus* species elsewhere in Japan. Collectively, the data suggest that *My. rufocanus* is the principal reservoir host species of PUUV in Japan and that spill-over events have occurred in *A. speciosus* and *A. argenteus*. 

We detected HTNV RNA in *N.* cf. *confucianus* in Vietnam. *N. confucianus* is widely distributed from the Indo-China Peninsula and adjacent mountains near southeastern Qinghai-Tibetan Plateau to Loess Plateau and northern China [41]. Recent genetic analysis of *Niviventer* species suggests that there are 16 species and one possible species complex. In addition, a morphologically similar species, *Chiromyscus*, is proposed to belong to *Niviventer.* The *Niviventer-Chiromyscus* complex is still unclear [42]. Our HTNV sequences, strains VN3973M589 and VN4004M620, were closely related to HTNV Da Bie Shan (DBS) strain that was detected in *N. confucianus* in Yunnan province, China [43]. The *CYTB* sequence of the host species of HTNV DBS strain was not reported in that study, but the results suggested that our newly detected strains (VN3973M589 and VN4004M620) and DBS were all harbored by *N. confucianus.*

In this research, we detected THAIV RNA in *R. rattus* captured in the Botanical and Zoological Garden of Tsimbazaza in Antananarivo, Madagascar. The same genotype of THAIV has been identified thus far [18,44]. Prototype THAIV was initially identified in *B. indica* in Thailand. Interestingly, the origin of Malagasy is constructed by Southeast Asia and East Africa based on Y-chromosomal sequences [45]. On the other hand, the house mouse, *Mus musculus*, in Madagascar is similar to the Yemen linage, suggesting that the Madagascar lineage was introduced from Yemen in Arabian Peninsula [46]. The precise origin of *R. rattus* in Madagascar is still unclear, but it may be associated with the peopling of Madagascar from Southeast Asia and Indonesia [47].

Co-circulation or host-sharing of rodent-borne hantaviruses has been reported for PUUV in bank voles (*My. glareolus*) [7], grey-sided voles (*My. rufocanus*) [8,9,10], and royal voles (*My. regulus*) [11,12] and for DOB/BGDV in yellow-necked field mice (*A. flavicollis*) [48], striped field mice (*A. agrarius*) [49], and Black Sea field mice (*A. ponticus*) [50]. Similar findings have been reported for non-rodent-borne hantaviruses, such as Seewis virus (SWSV) in the Eurasian shrew (*Sorex araneus*) [26], Laxmann’s shrew (*Sorex caecutiens*) [24], tundra shrew (*Sorex tundrensis*) [51], and Eurasian pygmy shrew (*Sorex minutus*) [52]. The present survey, showing the detection of PUUV infection in *A. speciosus* and *A. argenteus*, and a recent investigation of THAIV in *E. majori* [18] represent examples of spill-over or host-switching events. THAIV infection in *R. rattus* may also represent host switching from *B. indica*. Further investigations are warranted to better clarify the evolutionary origins and worldwide expansion of rodent-borne hantaviruses.

## Figures and Tables

**Table 1:** Summary of collected rodent samples.

Species	Japan							Madagascar		Myanmar				Vietnam			Total
	2011	2012	2013	2014	2015	2016	2017	2014	2015	2012	2013	2014	2015	2012	2013	2014	
*Apodemus argenteus*	43	13	62	18	30		2										168
*Apodemus speciosus*	43	9	87	7	29	1	13										189
*Bandicota bengalensis*											18	2	4				24
*Bandicota indica*												2			10		12
*Bandicota savilei*											4						4
*Berylmys berdmorei*															3		3
*Berylmys bowersi*														1	2	1	4
*Dacnomys millardi*															1		1
*Eothenomys andersoni*						2											2
*Eothenomys smithi*					4												4
*Eothenomys* sp.																1	1
*Leopoldamys sabanus*															1		1
*Maxomys moi*															1		1
*Maxomys surifer*														1	11		12
*Micromys minutus*						2											2
*Microtus montebelli*						1											1
*Mus caroli*												10			1		11
*Mus cookii*												1					1
*Mus fragilicauda*												1					1
*Mus lepidoides*													1				1
*Mus musculus*			9	4	25		1		7								46
*Mus musculus castaneus*	1							1			5		3				10
*Mus musculus musculus*									3								3
*Mus nitidulus*												11	3				14
*Mus pahari*															1		1
*Myodes rex*	1				1												2
*Myodes rufocanus bedfordiae*	5	7	27	3	36	3	12										93
*Myodes rutilus mikado*				1	1		4										6
*Niviventer cf. confucianus*															2	32	34
*Niviventer fulvescens*												1				1	2
*Niviventer* sp.																1	1
*Niviventer huang*															6		6
*Niviventer langbianis*															1		1
*Rattus andamanensis*														13			13
*Rattus exulans*											3	8	7		22		40
*Rattus nitidus*															2		2
*Rattus norvegicus*		28	26	4		2			5								65
*Rattus rattus* species complex	1	8	2			110	3	1	7			4					136
*Rattus tanezumi* species complex							44			1	4	5		5	1	1	61
*Rattus tiomanicus*															2		2
Total	94	65	213	37	126	121	79	2	22	1	34	45	18	20	67	37	981

## Data Availability

GenBank accession numbers for the PUUV sequences include strains KT3011KTF49 (S: MZ343341; M: MZ505035; L: MZ343342), KT3028KTF66 (S: MZ343343; M: MZ505036; L: MZ343344), KT3120KTF116 (S: MZ343345; L: MZ343346), KT3122KTF118 ( L: MZ343347), JA4032KTF597 (S: MZ343348; L: MZ343349), JA4277KTF-637 (M: MZ505037; L: MZ343350), JA5171KTF862 (S: MZ343351; L: MZ343352), JA5277KTF945 (S: MZ343355; L: MZ343356), JA6551KTF153 (S: MZ343357; M: MZ505038; L: MZ343358), UA1818B74 (S: MZ343339; M: MZ505034; L: MZ343340), JA6557KTF159 (M: MZ343359;), and JA5274KTF935 (S: MZ343353; L: MZ343354) from Japan; HTNV sequences include strains VN3973M589 (S: MZ343363; M: MZ343364; L: MZ343365) and VN4004M620 (S: MZ343366; M: MZ343367; L: MZ343368) from Vietnam; and THAIV sequences include strain MDG3887MG9 (S: MZ343360; M: MZ343361; L: MZ343362) from Madagascar. Additionally, host mitochondria sequences for *Myodes rufocanus bedfordiae* KT3011KTF49 (*COI*: MZ356210), KT3028KTF66 (*CYTB*: MZ356199; *COI*: MZ356211), KT3120KTF116 (*CYTB*: MZ356200; *COI*: MZ356212), KT3122KTF118 (*CYTB*: MZ356201; *COI*: MZ356213), JA4032KTF597 (*CYTB*: MZ356202; *COI*: MZ356214), JA4034KTF599 (*CYTB*: MZ356222), JA4277KTF-637 (*CYTB*: MZ356203; *COI*: MZ356215), JA5171KTF862 (*CYTB*: MZ356204; *COI*: MZ356216), JA5277KTF945 (*CYTB*: MZ356205; *COI*: MZ356217), and JA6551KTF153 (*CYTB*: MZ356206; *COI*: MZ356218),; *Apodemus speciosus* JA6557KTF159 (*CYTB*: MZ356207; *COI*: MZ356219); *Niviventer* cf. *confucianus* VN3973M589 (*CYTB*: MZ356208; *COI*: MZ356220); and VN4004M620 (*CYTB*: MZ356209; *COI*: MZ356221) and *Rattus rattus* MDG4084 (*CYTB*: MZ361584).

## Supplementary Materials

The following are available online at https://www.mdpi.com/article/10.3390/v13071343/s1. Supplementary Table S1: Oligonucleotide primers for amplification of the S, M, and L segments of soricine rodent-borne hantaviruses; Supplementary Table S2: GenBank accession numbers and host information for rodent-borne hantaviruses included in genetic and phylogenetic analysis; Table S3: GenBank accession numbers and species information for phylogenetic analysis.

## References

[1] Laenen L., Vergote V., Calisher C.H., Klempa B., Klingström J., Kuhn J.H., Maes P. (2019). *Hantaviridae*: Current classification and future perspectives. Viruses.

[2] Plyusnin A., Vapalahti O., Vaheri A. (1996). Hantaviruses: Genome structure, expression and evolution. J. Gen. Virol..

[3] Hepojoki J., Strandin T., Lankinen H., Vaheri A. (2012). Hantavirus structure—Molecular interactions behind the scene. J. Gen. Virol..

[4] Colleen B., Jonsson C.B., Figueiredo L.T.M., Vapalahti O. (2010). A global perspective on hantavirus ecology, epidemiology, and disease. Clin. Microbiol. Rev..

[5] Arai S., Yanagihara R. (2020). Genetic diversity and geographic distribution of bat-borne hantaviruses. Curr. Issues Mol. Biol..

[6] Lee H.W., Lee P.W., Johnson K.M. (1978). Isolation of the etiologic agent of Korean hemorrhagic fever. J. Infect. Dis..

[7] Brummer-Korvenkontio M., Vaheri A., Hovi T., von Bonsdorff C.H., Vuorimies J., Manni T., Penttinen K., Oker-Blom N., Lähdevirta J. (1980). Nephropathia epidemica: Detection of antigen in bank voles and serologic diagnosis of human infection. J. Infect. Dis..

[8] Kariwa H., Yoshizumi S., Arikawa J., Yoshimatsu K., Takahashi K., Takashima I., Hashimoto N. (1995). Evidence for the existence of Puumula-related virus among *Clethrionomys rufocanus* in Hokkaido, Japan. Am. J. Trop. Med. Hyg..

[9] Sanada T., Seto T., Ozaki Y., Saasa N., Yoshimatsu K., Arikawa J., Yoshii K., Kariwa H. (2012). Isolation of Hokkaido virus, genus Hantavirus, using a newly established cell line derived from the kidney of the grey red-backed vole (*Myodes rufocanus bedfordiae*). J. Gen. Virol..

[10] Yashina L.N., Abramov S.A., Dupal T.A., Danchinova G.A., Malyshev B.S., Hay J., Gu S.H., Yanagihara R. (2015). Hokkaido genotype of Puumala virus in the grey red-backed vole (*Myodes rufocanus*) and northern red-backed vole (*Myodes rutilus*) in Siberia. Infect. Genet. Evol..

[11] Song K.J., Baek L.J., Moon S., Ha S.J., Kim S.H., Park K.S., Klein T.A., Sames W., Kim H.C., Lee J.S. (2007). Muju virus, a novel hantavirus harboured by the arvicolid rodent *Myodes regulus* in Korea. J. Gen. Virol..

[12] Lee J.G., Gu S.H., Baek L.J., Shin O.S., Park K.S., Kim H.C., Klein T.A., Yanagihara R., Song J.-W. (2014). Muju virus, harbored by *Myodes regulus* in Korea, might represent a genetic variant of Puumala virus, the prototype arvicolid rodent-borne hantavirus. Viruses.

[13] Elwell M.R., Ward G.S., Tingpalapong M., Leduc J.W. (1985). Serologic evidence of Hantaan-like virus in rodents and man in Thailand. S. Asian J. Trop. Med. Public Health.

[14] Hugot J.P., Plyusnina A., Herbreteau V., Nemirov K., Laakkonen J., Lundkvist A., Supputamongkol Y., Henttonen H., Plyusnin A. (2006). Genetic analysis of Thailand hantavirus in *Bandicota indica* trapped in Thailand. Virol. J..

[15] Pattamadilok S., Lee B.H., Kumperasart S., Yoshimatsu K., Okumura M., Nakamura I., Araki K., Khoprasert Y., Dangsupa P., Panlar P. (2006). Geographical distribution of hantaviruses in Thailand and potential human health significance of Thailand virus. Am. J. Trop. Med. Hyg..

[16] Plyusnina A., Ibrahim I.N., Plyusnin A. (2009). A newly recognized hantavirus in the Asian house rat (*Rattus tanezumi*) in Indonesia. J. Gen. Virol..

[17] Johansson P., Yap G., Low H.T., Siew C.C., Kek R., Ng L.C., Bucht G. (2010). Molecular characterization of two hantavirus strains from different rattus species in Singapore. Virol. J..

[18] Reynes J.M., Razafindralambo N.K., Lacoste V., Olive M.M., Barivelo T.A., Soarimalala V., Heraud J.M., Lavergne A. (2014). Anjozorobe hantavirus, a new genetic variant of Thailand virus detected in rodents from Madagascar. Vector Borne Zoonotic Dis..

[19] Ramsden C., Holmes E.C., Charleston M.A. (2009). Hantavirus evolution in relation to its rodent and insectivore hosts: No evidence for codivergence. Mol. Biol. Evol..

[20] Bennett S.N., Gu S.H., Kang H.J., Arai S., Yanagihara R. (2014). Reconstructing the evolutionary origins and phylogeography of hantaviruses. Trends Microbiol..

[21] Guterres A., de Oliveira R.C., Fernandes J., de Lemos E.R.S. (2019). The mystery of the phylogeographic structural pattern in rodent-borne hantaviruses. Mol. Phylogenet. Evol..

[22] Sikes R.S. (2016). Animal Care and Use Committee of the American Society of Mammalogists. 2016 Guidelines of the American Society of Mammalogists for the use of wild mammals in research and education. J. Mammal..

[23] Kirkland G.L. (1998). Guidelines for the Capture, Handling, and Care of Mammals as Approved by the American Society of Mammalogists. J. Mammal..

[24] Arai S., Kang H.J., Gu S.H., Ohdachi S.D., Cook J.A., Yashina L.N., Tanaka-Taya K., Abramov S.A., Morikawa S., Okabe N. (2016). Genetic diversity of Artybash virus in the Laxmann’s shrew (*Sorex caecutiens*). Vector Borne Zoonotic Dis..

[25] Arai S., Ohdachi S.D., Asakawa M., Kang H.J., Mocz G., Arikawa J., Okabe N., Yanagihara R. (2008). Molecular phylogeny of a newfound hantavirus in the Japanese shrew mole (*Urotrichus talpoides*). Proc. Natl. Acad. Sci. USA.

[26] Song J.-W., Gu S.H., Bennett S.N., Arai S., Puorger M., Hilbe M., Yanagihara R. (2007). Seewis virus, a genetically distinct hantavirus in the Eurasian common shrew (*Sorex araneus*). Virol. J..

[27] Klempa B., Fichet-Calvet E., Lecompte E., Auste B., Aniskin V., Meisel H., Barriere P., Koivogui L., ter Meulen J., Krüger D.H. (2007). Novel hantavirus sequences in shrew, Guinea. Emerg. Infect. Dis..

[28] Klempa B., Fichet-Calvet E., Lecompte E., Auste B., Aniskin V., Meisel H., Denys C., Koivogui L., ter Meulen J., Krüger D.H. (2006). Hantavirus in African wood mouse, Guinea. Emerg. Infect. Dis..

[29] Song J.-W., Kang H.J., Song K.J., Truong T.T., Bennett S.N., Arai S., Truong N.U., Yanagihara R. (2007). Newfound hantavirus in Chinese mole shrew, Vietnam. Emerg. Infect. Dis..

[30] Arai S., Taniguchi S., Aoki K., Yoshikawa Y., Kyuwa S., Tanaka-Taya K., Masangkay J.S., Omatsu T., Puentespina R., Watanabe S. (2016). Molecular phylogeny of a genetically divergent hantavirus harbored by the Geoffroy’s rousette (*Rousettus amplexicaudatus*), a frugivorous bat species in the Philippines. Infect. Genet. Evol..

[31] Kikuchi F., Aoki K., Ohdachi S.D., Tsuchiya K., Motokawa M., Jogahara T., Son N.T., Bawm S., Lin K.S., Thwe T.L. (2020). Genetic diversity and phylogeography of Thottapalayam thottimvirus (*Hantaviridae*) in Asian house shrew (*Suncus murinus*) in Eurasia. Front. Cell. Infect. Microbiol..

[32] Thompson J.D., Higgins D.G., Gibson T.J. (1994). CLUSTAL W: Improving the sensitivity of progressive multiple sequence alignment through sequence weighting, position-specific gap penalties and weight matrix choice. Nucleic Acids Res..

[33] Tippmann H.F. (2004). Analysis for free: Comparing programs for sequence analysis. Brief. Bioinform..

[34] Ronquist F., Huelsenbeck J.P. (2003). MrBayes 3: Bayesian phylogenetic inference under mixed models. Bioinformatics.

[35] Posada D., Crandall K.A. (1998). Modeltest: Testing the model of DNA substitution. Bioinformatics.

[36] Darriba D., Taboada G.L., Doallo R., Posada D. (2012). jModelTest 2: More models, new heuristics and parallel computing. Nat. Methods.

[37] Jackson A.P., Charleston M.A. (2004). A cophylogenetic perspective of RNA-virus evolution. Mol. Biol. Evol..

[38] Charleston M.A., Robertson D.L. (2002). Preferential host switching by primate lentiviruses can account for phylogenetic similarity with the primate phylogeny. Syst. Biol..

[39] Lokugamage N., Kariwa H., Lokugamage K., Iwasa M.A., Hagiya T., Yoshii K., Tachi A., Ando S., Fukushima H., Tsuchiya K. (2004). Epizootiological and epidemiological study of hantavirus infection in Japan. Microbiol. Immunol..

[40] Lee B.H., Yoshimatsu K., Araki K., Ogino M., Okumura M., Tsuchiya K., Kariwa H., Arikawa J. (2003). Detection of antibody for the serodiagnosis of hantavirus infection in different rodent species. Arch. Virol..

[41] Ge D., Lu L., Abramov A.V., Wen Z., Cheng J., Xia L., Vogler A.P., Yang Q. (2019). Coalescence models reveal the rise of the white-bellied rat *(Niviventer confucianus*) following the loss of Asian megafauna. J. Mammal. Evol..

[42] Balakirev A.E., Abramov A.V., Rozhnov V.V. (2014). Phylogenetic relationships in the *Niviventer*-*Chiromyscus* complex (*Rodentia*, *Muridae*) inferred from molecular data, with description of a new species. ZooKeys.

[43] Cao Z.W., Zuo S.Q., Gong Z.D., Zhan L., Bian C.L., Zhang P.H., Yang H., Zhang J.S., Zhao Q.M., Jia N. (2010). Genetic analysis of a hantavirus strain carried by *Niviventer confucianus* in Yunnan province, China. Virus Res..

[44] Raharinosy V., Olive M.M., Andriamiarimanana F.M., Andriamandimby S.F., Ravalohery J.P., Andriamamonjy S., Filippone C., Rakoto D.A.D., Telfer S., Heraud J.M. (2018). Geographical distribution and relative risk of Anjozorobe virus (Thailand orthohantavirus) infection in black rats (*Rattus rattus*) in Madagascar. Virol. J..

[45] Hurles M.E., Sykes B.C., Jobling M.A., Forster P. (2005). The dual origin of the Malagasy in Island Southeast Asia and East Africa: Evidence from maternal and paternal lineages. Am. J. Hum. Genet..

[46] Sakuma Y., Ranorosoa M.C., Kinoshita G., Shimoji H., Tsuchiya K., Ohdachi S.D., Arai S., Tanaka C., Ramino H., Suzuki H. (2016). Variation in the coat-color-controlling genes, *Mc1r* and *Asip*, in the house mouse *Mus musculus* from Madagascar. Mammal Study.

[47] Heiske M., Alva O., Pereda-Loth V., Van Schalkwyk M., Radimilahy C., Letellier T., Rakotarisoa J.-A., Pierron D. (2021). Genetic evidence and historical theories of the Asian and African origins of the present Malagasy population. Hum. Mol. Genet..

[48] Xiao S.Y., Diglisic G., Avšič-Županc T., LeDuc J.W. (1993). Dobrava virus as a new hantavirus: Evidenced by comparative sequence analysis. J. Med. Virol..

[49] Plyusnina A., Ferenczi E., Racz G.R., Nemirov K., Lundkvist A., Vaheri A., Vapalahti O., Plyusnin A. (2009). Co-circulation of three pathogenic hantaviruses: Puumala, Dobrava, and Saaremaa in Hungary. J. Med. Virol..

[50] Dzagurova T.K., Witkowski P.T., Tkachenko E.A., Klempa B., Morozov V.G., Auste B., Zavora D., Iunicheva I.V., Mutnih E.S., Krüger D.H. (2012). Isolation of Sochi virus from a fatal case of hantavirus disease with fulminant clinical course. Clin. Infect. Dis..

[51] Yashina L.N., Abramov S.A., Gutorov V.V., Dupal T.A., Krivopalov A.V., Panov V.V., Danchinova G.A., Vinogradov V.V., Luchnikova E.M., Hay J. (2010). Seewis virus: Phylogeography of a shrew-borne hantavirus in Siberia, Russia. Vector Borne Zoonotic Dis..

[52] Gu S.H., Hejduk J., Markowski J., Kang H.J., Markowski M., Połatyńska M., Sikorska B., Liberski P.P., Yanagihara R. (2014). Co-circulation of soricid- and talpid-borne hantaviruses in Poland. Infect. Genet. Evol..

