# Supplementary material for: Rodent-Borne Orthohantaviruses in Vietnam, Madagascar and Japan
Source: Viruses. 2021 Jul 12;13(7):1343. doi: 10.3390/v13071343 (PMC8310111; doi:10.3390/v13071343)
Supplement: Supplementary file 1 [file viruses-13-01343-s001.zip › TableS/Supplementary Tables1.pdf]

Supplementary Table S1: Oligonucleotide primers for amplification of the S, M and L segments of rodent-borne hantaviruses

Primer	Segment	5' to 3'	Polarity
OSM55	S/M/L	TAGTAGTAGACTCC	+/-
PHS-5endF	S	TAGTAGTAGACTCCTTRAARAGC	+
S437F	S	SWGGTCARACTGCHRAYTGG	+
H4S-509R	S	AATCCTTGTCCCTTTGTTGTCC	-
Cro2F	S	AGYCCIGTIATGRGWGTIRTYGG	+
HTN-S4	S	GAIIGITGTCCACCAACATG	-
HTS-1032F	S	CAGGIATGGCAGARYTIGGIGC	+
Cro2R	S	AIGAYTGRTARAAIGAIGAYTTYTT	-
JJUVS-1233R	S	TCACCMAGRTGRAAGTGRTCIAC	-
HTN-S6	S	AGCTCIGGATCCATITCATC	-
SAS-1248F	S	CAYCTKGGTGAYGATATGGAYCC	+
HTS-3R	S	TAGTAGTAIGCTCCYT	-
Rm-225F	M	CCYGARAGTTCTTGTAAYATGG	+
MG373M-541R	M	GCKACAATRCARCTYTTCATC	-
OSV697F	M	GGACCAGGTGCADCTTGTGAAGC	+
MKWM-917R	M	TRTCATGATCTTCICCATRTGG	-
PUUM-1064F	M	GATGAYCCTGTRTATRTITGGGC	+
T-M1199F	M	TAAVTTCAMCAACATGTCT	+
COM-1257R	M	CCICCYTCWGCAAAIGCITCACA	-
PUUM-1331R	M	TYAYTTGYTGYTCWGCACCACG	-
T-M1485R	M	CCAGCCAAARCARAATGT	-
HTM-1490F	M	TGTGTICCWGGITTYCATGGIT	+
PUUM-2064R	M	CCWGADCCATGKGCWGTATCWG	-
SHM-2371F	M	TGYAACCCIGTIGATTGYCCWGG	+
HTM-2409R	M	CCACAIGCWGTRCAICCWGT	-
MKWM-2631R	M	CATGATRTCICCAGGRTCICC	-
TM-2957R	M	GAACCCCADGCCCCITCYAT	-
SMGM-3142F	M	AGRGGTGGACATAGTGGSTC	+
HTM-3211R	M	CCRSWATGICCICCTTTICC	-
PHM-3endR	M	TAGTAGTAGACTCCGCAAGAA	+/-
HNL-5F	L	TAGTAGTAKACTCCSKRA	+
SL-513F	L	GTIGTIGCAGTIAGGACWGATGG	+
PUUL-854F	L	GGGAGGARGAAGARATMCATGC	+
CS-L-2319R	L	CTTCYTCATTYACATTMCCATG	-
PUUL-2332R	L	TYCTDGCCCAYTCAACYGTYTC	+
HAN-L-F1	L	ATGTAYGTBAGTGCWGATGC	+
HAN-L-F2	L	TGCWGATGCHACIAARTGGTC	+
HTL-2971R	L	CWGGWGACCAYTTIGTDGCATC	-
HAN-L-R2	L	GCRTCRTCWGARTGRTGDGCAA	-
HAN-L-R1	L	AACCADTCWGTYCCRTCATC	-
RL-3881R	L	ATRGACATKGCTCCACTKCCAC	-
RL-4058R	L	CGITCATGTYGAAAIRTNTCATCAGA	-
HNL-4773F	L	RGGDACRGCWGAAGCWATWTGTG	+
CR-L-5461F	L	CTGAIKCWGGITTTGARTGGGG	+
CR-L-6339R	L	GAATCAGGWARKACATCTTCHGG	-
HNL-3R	L	TAGTAGTAKGCTCCG	-

Abbreviations: A, Adenine; B, C or G or T; C, Cytosine; D, A or G or T; G, Guanine; H, A or C or T; I, Inosine; K, G or T; M, A or C; N, any nucleotide; R, A or G; S, G or C; T, Thymine; V, A or C or G; W, A or T; Y, C or T.
