# Supplementary material for: Rodent-Borne Orthohantaviruses in Vietnam, Madagascar and Japan
Source: Viruses. 2021 Jul 12;13(7):1343. doi: 10.3390/v13071343 (PMC8310111; doi:10.3390/v13071343)
Supplement: Supplementary file 1 [file viruses-13-01343-s001.zip › TableS/Supplementary TablesR3.pdf]

Supplementary Table S3. GenBank accession numbers and species information for phylogenetic analysis.

Soecies	Clone	Country	*CYTB* accession number
Suncus murinus	NP6362	Nepal	MT344840
Hipposideros pomona	VN1982B4	Vietnam	JX912954
Eliurus majori		Madagascar	AF160549
Stenocephalemys albipes		Ethiopia	AF518346
Hylomyscus simus		Guinea	DQ212188
Apodemus flavicollis		Greece	JF819967
Apodemus peninsulae		South Korea	AB073811
Apodemus agrarius		South Korea	AB303225
Apodemus argenteus	JA5274KTF935	Japan	MK410343
Apodemus speciosus	UA1818B74	Japan	MK410319
Apodemus speciosus	JA6557KTF159	Japan	MZ356207
Rattus norvegicus		South Korea	AB355903
Bandicota indica		Thailand	KJ592782
Rattus rattus	MDG3887MG9	Madagascar	LC147016
Rattus rattus	MDG4084	Madagascar	MZ361584
Niviventer cf. *confucianus*	VN3973	Vietnam	MZ356208
Niviventer cf. *confucianus*	VN4004	Vietnam	MZ356209
Niviventer confucianus		China	KY304471
Myodes regulus		South Korea	NC_016427
Myodes rufocanus bedfordiae	KT3011KTF49	Japan	LC406450
Myodes rufocanus bedfordiae	KT3028KTF66	Japan	MZ356199
Myodes rufocanus bedfordiae	KT3120KTF116	Japan	MZ356200
Myodes rufocanus bedfordiae	JA4032KTF597	Japan	MZ356202
Myodes rufocanus bedfordiae	JA4034KTF599	Japan	MZ356222
Myodes rufocanus bedfordiae	JA4277KTF-637	Japan	MZ356203
Myodes rufocanus bedfordiae	JA5171KTF862	Japan	MZ356204
Myodes rufocanus bedfordiae	KT5277KTF945	Japan	MZ356205
Myodes rufocanus bedfordiae	JA6551KTF153	Japan	MZ356206
Myodes rufocanus bedfordiae	Kamiiso	Japan	LC416891
Myodes rufocanus bedfordiae	Kitahiyama	Japan	AB675420
Myodes rufocanus	MrOlhon109	Russia	KP859520
Myodes rufocanus	AF15465	Russia	AY309415
Myodes glareolus		Finland	JX477304
Microtus oeconomus		Russia	AY305050
Microtus arvalis		Finland	AY220770
Microtus obscurus		China	MN183143
Microtus pennsylvanicus		US	AF119279
Microtus californicus		US	AF163891
Oligoryzomys longicaudatus		Chile	AF346566
Peromyscus maniculatus		US	AF119261
Eothenomys eleusis	FG10	China	KT899700
Sigmodon alstoni	T2140	Venezuela	AF293396
Microtus oeconomus	3277_McCarthy		AY305153
Microtus agrestis	England 5	England	AY167191
Microtus agrestis	Germany 4	Germany	GU563294
