# Supplementary material for: Rodent-Borne Orthohantaviruses in Vietnam, Madagascar and Japan
Source: Viruses. 2021 Jul 12;13(7):1343. doi: 10.3390/v13071343 (PMC8310111; doi:10.3390/v13071343)
Supplement: Supplementary file 1 [file viruses-13-01343-s001.zip › TableS/Supplementary TablesRS2.pdf]

Supplementary Table S2. GenBank accession numbers and host information for rodent-borne hantaviruses included in genetic and phylogenetic analysis.

Virus Name	Strain Name	Country	S segment length for analysis accession no.	M segment length for analysis accession no.	L segment length for analysis accession no.	Host species	*CYTB* accession no.	*COI* accession no.
Hantaan orthohantavirus	VN3973M589	Vienam	1290-nt MZ343363	2922-nt MZ343364	6456-nt MZ343365	*Niviventer* cf. *confucianus*	MZ356208	MZ356220
Hantaan orthohantavirus	VN4004M620	Vietnam	1290-nt MZ343366	3212-nt MZ343367	6456-nt MZ343368	*Niviventer* cf. *confucianus*	MZ356209	MZ356221
Thailand orthohantavirus	MDG3887MG9	Madagascar	1290-nt MZ343360	3402-nt MZ343361	6456-nt MZ343362	*Rattus rattus*	LC147016	MK410392
Puumala orthohantavirus	JA3011KTF49	Japan	240-nt MZ343341	223-nt MZ505035	2429-nt MZ343342	*Myodes rufocanus bedfordiae*	LC406450	MZ356210
Puumala orthohantavirus	JA3028KTF66	Japan	237-nt MZ343343	223-nt MZ505036	2566-nt MZ343344	*Myodes rufocanus bedfordiae*	MZ356199	MZ356211
Puumala orthohantavirus	JA3120KTF116	Japan	235-nt MZ343345	ND	3602-nt MZ343346	*Myodes rufocanus bedfordiae*	MZ356200	MZ356212
Puumala orthohantavirus	JA3122KTF118	Japan	ND	ND	349-nt MZ343347	*Myodes rufocanus bedfordiae*	MZ356201	MZ356213
Puumala orthohantavirus	JA4032KTF597	Japan	831-nt MZ343348	ND	1169-nt MZ343349	*Myodes rufocanus bedfordiae*	MZ356202	MZ356214
Puumala orthohantavirus	JA4277KTF-637	Japan	ND	223-nt MZ505037	349-nt MZ343350	*Myodes rufocanus bedfordiae*	MZ356203	MZ356215
Puumala orthohantavirus	JA5171KTF862	Japan	220-nt MZ343351	ND	349-nt MZ343352	*Myodes rufocanus bedfordiae*	MZ356204	MZ356216
Puumala orthohantavirus	JA5277KTF945	Japan	231-nt MZ343355	ND	349-nt MZ343356	*Myodes rufocanus bedfordiae*	MZ356205	MZ356217
Puumala orthohantavirus	JA6551KTF153	Japan	704-nt MZ343357	1275-nt MZ505038	1011-nt MZ343358	*Myodes rufocanus bedfordiae*	MZ356206	MZ356218
Puumala orthohantavirus	UA1818B74	Japan	697-nt MZ343339	223-nt MZ505034	2596-nt MZ343340	*Apodemus speciosus*	MK410319	MK410366
Puumala orthohantavirus	JA6557KTF159	Japan	ND	824-nt MZ343359	ND	*Apodemus speciosus*	MZ356207	MZ356219
Puumala orthohantavirus	JA5274KTF935	Japan	808-nt MZ343353	ND	375-nt MZ343354	*Apodemus argenteus*	MK410343	MK410423

ND: not detected
